# Periprosthetic Joint Infection

**DOI:** 10.1155/2019/6834680

**Published:** 2019-01-13

**Authors:** Bernd Fink, Konstantinos Anagnostakos, Heinz Winkler

**Affiliations:** ^1^Department of Joint Replacement, General and Rheumatic Orthopaedics, Orthopaedic Clinic Markgröningen gGmbH, Kurt-Lindemann-Weg 10, 71706 Markgröningen, Germany; ^2^Orthopaedic Department, University-Hospital Hamburg-Eppendorf, Martinistrasse 52, 20246 Hamburg, Germany; ^3^Orthopaedic Department, Klinikum Saarbrücken, Winterberg 1, 66119 Saarbrücken, Germany; ^4^PremiQaMed Privatkliniken GmbH Heiligenstädter Straße 55-63, 1190 Wien, Austria

The periprosthetic infection (PPI) of hip, knee, and shoulder endoprostheses is, with an incidence of around 1%, an uncommon but nevertheless devastating complication of arthroplasty procedures [1, 2]. The classification proposed by Tsukayama et al. [3] differentiates between acute early and chronic late infections whereby the threshold between the two is 4 weeks after the surgical intervention. However, other authors regard infections occurring up to 3 months after surgery as early infections [4, 5]. Acute periprosthetic infections that arise after many trouble-free years as a result of an infection at a remote site are classified as acute hematogenous infections and are treated in the same way as acute early infections [3].

When early infections occur, within 4 weeks of implantation, the implant can be left in place with a high probability of cure whereas late infections require prosthesis revision to eradicate the infection [6]. In such cases, one can differentiate between one-stage and two-stage revisions. Two-stage revision involves an initial operation to remove all foreign materials and this is followed by an interim phase of mostly 6–12 weeks, either left as a Girdlestone situation or with the implantation of a cement spacer.

Whereas early infections, i.e., those occurring within the first four weeks of implantation, usually cause local and systemic inflammatory reactions, these are often missing in cases of late periprosthetic joint infection with low-grade symptoms, occurring later than four weeks after implantation [3]. This makes the diagnosis of late periprosthetic infections very much more difficult. The classical clinical signs, laboratory tests, and imaging techniques such as X-ray and scintigraphy are associated with a high level of false positives and false negatives [7].

A preoperative diagnostic before revision surgery takes place is helpful because therapeutic strategy differs in septic revisions from aseptic revision; local and systemic antibiotic therapy can be planned specifically before surgery takes place and can be started at a time before new biofilm formation on a new prosthesis has taken place [1, 2].

There are many questions pertaining to both the diagnostic of periprosthetic joint infection (PJI) and its treatment and existing procedures are based more on empirical findings than on data from prospective studies with a high level of evidence. This special issue on periprosthetic joint infection discusses important details in the diagnostic and therapeutic procedures.

Even though the detection of the microorganism causing the periprosthetic joint infection is the most important diagnostic tool, the paper of D. Karczewski et al. shows that the indication for a septic revision can also solely be based on the intraoperative (para-)clinical signs fistula or purulence, Krenn–Morawietz histological type 2 or 3, and joint aspirate > 2000/*μ*l leukocytes or >70% granulocytes. The paper of G. Bori et al. gives an update about the histopathology in periprosthetic joint infection. The paper of S.-J. Lim et al. underline that the preoperative CRP on its own does not have a strong power for the diagnostic of PJI by showing that patients with a hip fracture and an elevated CRP have higher CRP-levels also postoperative compared to those with normal CRP values without having a higher risk for PJI. S. P. Boelch et al. show in their paper that the aspiration of the joint with a spacer in a two-stage procedure of an infected total knee arthroplasty for cultivation of the aspirate is not helpful for the decision whether an reimplantation can be done or not.

For the topic of treatment of PJI the other paper of S. P. Boelch et al. reported that Copal® cement and Palacos® R+G cement for the use as gentamicin and vancomycin biantibiotic-loaded spacer have comparable elution levels of the antibiotics out of the spacers. D. H. Ro et al. could show that periprosthetic joint infection does not preclude good clinical outcomes after a revision total knee arthroplasty. However, poor outcomes were mainly associated with large bone defects and an increased number of previous surgeries. In a systemic review and meta-analysis M. Reisener and C. Perka found out that culture-negative PJIs have comparable outcomes than culture-positive PJIs. However, B. Zatorska et al. could show that the production of extracellular DNA of* Staphylococcus epidermidis* in 24 hours biofilms correlates with the patients' outcome “not cured” after 12 months. However, for* Staphylococcus aureus* infections no such correlation was detected. If two-stage revisions failed M. Faschingbauer et al. detected that irrigation and debridement have a chance of 63.2% of success and may therefore be an therapeutical option for acute reinfections after failed two-stage revisions if performed within the first 30 postoperative days or if symptoms are present for less than 3 weeks. For the reimplantation in two-stage septic revisions F. Reichel et al. showed that tranexamic acid is effective for the reduction of blood loss.

B. Fink and F. Sevelda worked out the specific diagnostic and therapeutic particularities for periprosthetic joint infections of the shoulder.

## References

[1] Fink B., Makowiak C., Fuerst M., Berger I., Schäfer P., Frommelt L. (2008). The value of synovial biopsy, joint aspiration and C-reactive protein in the diagnosis of late peri-prosthetic infection of total knee replacements. *The Journal of Bone & Joint Surgery (British Volume)*.

[2] Fink B., Lass R. (2014). A diagnostic algorithm for painful total knee replacement. *Z Orthop Unfall*.

[3] Tsukayama D. T., Estrada R., Gustilo R. B. (1996). Infection after total hip arthroplasty: a study of the treatment of one hundred and six infections. *The Journal of Bone & Joint Surgery*.

[4] Martínez-Pastor J. C., Muñoz-Mahamud E., Vilchez F. (2009). Outcome of acute prosthetic joint infections due to gram-negative bacilli treated with open debridement and retention of the prosthesis. *Antimicrobial Agents and Chemotherapy*.

[5] Aboltins C. A., Dowsey M. M., Buising K. L. (2011). Gram-negative prosthetic joint infection treated with debridement, prosthesis retention and antibiotic regimens including a fluoroquinolone. *Clinical Microbiology and Infection*.

[6] Cui Q., Mihalko W. M., Shields J. S., Ries M., Saleh K. J. (2007). Antibiotic-Impregnated Cement Spacers for the Treatment of Infection Associated with Total Hip or Knee Arthroplasty. *The Journal of Bone and Joint Surgery-American Volume*.

[7] Trampuz A., Zimmerli W. (2005). Prosthetic joint infections: update in diagnosis andtreatment. *Swiss Med Wkly*.

